# Increased progression free survival with radiolabelled somatostatin receptor antagonist versus standard peptide receptor radionuclide therapy in patients with somatostatin receptor positive tumours

**DOI:** 10.1007/s00259-026-07856-9

**Published:** 2026-03-24

**Authors:** Abdulrafee Mushaweh, Christopher Eigler, Lisa McDougall, Andreas Bauman, Emanuel Christ, Peter Bernhardt, Michael Hentschel, Guillaume Nicolas, Melpomeni Fani, Damian Wild

**Affiliations:** 1 Clinic for Radiology and Nuclear Medicine, University Hospital Basel, Petersgraben 4, Basel, CH-4031 Switzerland; 2 Department of Medical Radiation Sciences, Institution of Clinical Science, University of Gothenburg, Gothenburg, Sweden; 3 ENETS Centre of Excellence for Neuroendocrine and Endocrine Tumours, University Hospital Basel, Basel, Switzerland; 4 Department of Endocrinology, University Hospital Basel, Basel, Switzerland

**Keywords:** Somatostatin receptor, Neuroendocrine tumours, Peptide receptor radionuclide therapy, DOTA-JR11, DOTA-TOC

## Abstract

**Purpose:**

Peptide Receptor Radionuclide Therapy (PRRT) with [^177^Lu]Lu-DOTA-TOC or [^177^Lu]Lu-DOTA-TATE provides limited response durability in neuroendocrine tumours (NETs). The somatostatin receptor subtype 2 (SSTR2) antagonist [^177^Lu]Lu-DOTA-JR11 may deliver higher tumour absorbed doses and enhanced efficacy. This study compared median progression-free survival (PFS) and disease control rate (DCR) after [^177^Lu]Lu-DOTA-JR11 rechallenge versus prior [^177^Lu]Lu-DOTA-TOC in the same patients with early-progressing metastatic NET.

**Methods:**

In this retrospective, single-centre compassionate-use study, 15 consecutive patients with metastatic NET who progressed ≤ 12 months after standard [^177^Lu]Lu-DOTA-TOC therapy (2–4 cycles; 5.6–7.5 GBq/cycle) received rechallenge PRRT: 1–2 cycles [^177^Lu]Lu-DOTA-TOC followed by 1–2 cycles [^177^Lu]Lu-DOTA-JR11 (2 GBq/m² body surface area; 2.8–4.8 GBq/cycle) at 10 ± 1 week intervals. Endpoints included comparison of median PFS, DCR, safety (CTCAE v5.0), and absorbed doses.

**Results:**

Median PFS was 11 months (12-month DCR 20%) after [^177^Lu]Lu-DOTA-TOC and 14 months (12-month DCR 67%) after rechallenge with [^177^Lu]Lu-DOTA-TOC + [^177^Lu]Lu-DOTA-JR11 (HR ~ 0.34, *p* = 0.007) in the same patients. Grade 3 adverse events were reversible, including lymphopenia/hypoalbuminemia ([^177^Lu]Lu-DOTA-TOC) and anaemia/lymphopenia ([^177^Lu]Lu-DOTA-JR11); no grade ≥ 4 events occurred. Median absorbed doses (Gy/cycle) were 18.5 (tumour), 2.2 (kidney), and 0.54 (bone marrow) for [^177^Lu]Lu-DOTA-JR11 versus 12.9, 2.0, and 0.21 for [^177^Lu]Lu-DOTA-TOC. Tumour-to-bone marrow ratios were comparable for both compounds.

**Conclusion:**

[^177^Lu]Lu-DOTA-JR11 achieved ~ 2-fold higher tumour absorbed doses and improved median PFS by 3 months (95% CI 1.4–10.8) in our cohort of early-progressing NET patients. These preliminary findings support further prospective evaluation of [^177^Lu]Lu-DOTA-JR11 in NET patients.

**Supplementary Information:**

The online version contains supplementary material available at https://doi.org/10.1007/s00259-026-07856-9.

## Introduction

Neuroendocrine neoplasms (NENs) encompass a diverse group of tumours, most frequently arising within the gastroenteropancreatic (GEP) tract. They are clinically stratified in secreting and non-secreting and histologically according to their proliferation index (Ki67) into well-differentiated neuroendocrine tumours (NETs) G1, G2 and G3 and poorly differentiated G3 neuroendocrine carcinomas (NECs, either small or large cell) [1]. NENs are often detected late, in particular if they are non-secreting as symptoms can be subtle or nonspecific. In this case curative R0 surgical resection is not possible anymore and systemic palliative treatment to control the disease is necessary.

Most NETs express somatostatin receptor subtype 2 (SST2) at high density which act as a target for peptide receptor radionuclide therapy (PRRT) [2]. PRRT with SST2 agonists [^177^Lu]Lu-DOTA-TOC ([^177^Lu]Lu-edotreotide) and [^177^Lu]Lu-DOTA-TATE ([^177^Lu]Lu-oxodotreotide) has been employed as second- or third-line therapy for patients with inoperable, advanced GEP-NETs (G1-2) [3, 4]. Although PRRT with [^177^Lu]Lu-DOTA-TOC is efficient with a median PFS of 24 months in PRRT naïve GEP NETs patients (G1-2), it is not curative [5]. Retrospective real-world evaluation of initial PRRT with ~ 4 cycles [^177^Lu]Lu-DOTA-TATE revealed a median PFS of 20.2 months in PRRT naïve NET patients [6]. Rechallenge PRRT with ~ 2 cycles [^177^Lu]Lu-DOTA-TATE in the same patient group showed a median PFS of 9.6 months and an overall survival of 12.6 months. Thus, there is an unmet need to improve rechallenge PRRT of patients with advanced NETs with more effective radiopharmaceuticals.

Ginj et al. hypothesized that radiolabelled SST antagonists may perform better than agonists [7]. In the meantime, there is compelling evidence that lutetium-177 labelled SST2 antagonists (e.g. [^177^Lu]Lu-DOTA-JR11 = [^177^Lu]Lu-OPS201 = [^177^Lu]Lu-satoreotide tetraxetan) binds to many more SST2 binding sites on the cell surface resulting in a higher tumour absorbed doses [8]. A clinical pilot study involving four patients with NET found that the SSTR2 antagonist [^177^Lu]Lu-DOTA-JR11 delivered higher tumour doses and more favourable tumour-to-kidney and tumour-to-bone marrow ratios compared to [^177^Lu]Lu-DOTA-TATE [9]. The most relevant findings of this study were 3.5-fold higher median tumour absorbed dose per GBq, more than 2-fold higher tumour-to-bone marrow absorbed dose ratio with [^177^Lu]Lu-DOTA-JR11 compared to [^177^Lu]Lu-DOTA-TATE and moderate adverse events with one grade 3 thrombocytopenia after treatment with 3 x ~ 5 GBq (total 15.2 GBq) of [^177^Lu]Lu-DOTA-JR11. Phase I and II studies could confirm the high absorbed tumour dose of radiolabelled SSTR2-antagonists translating into very promising first efficacy data [10–12]. Despite these promising observations, no clinical trial has evaluated therapy with a radiolabelled SSTR2-antagonist in NET patients who progress following standard treatment with a radiolabelled SSTR2-agonist. The primary aim of this study is to compare median PFS, 12 months DCR and safety of [^177^Lu]Lu-DOTA-JR11 (SSTR2 antagonist) with our standard treatment, [^177^Lu]Lu-DOTA-TOC (SSTR2 agonist). A secondary objective is to compare tumour and organ dosimetry of both compounds.

## Materials and methods

### Study design and patients

Patients were consecutively recruited for this preplanned compassionate use, retrospective, open label, single centre, single-arm, intra-patient comparison study. The main inclusion criteria were histologically confirmed neuroendocrine tumour WHO grade 1–3 with progression ≤ 12 months after termination of standard PRRT with [^177^Lu]Lu-DOTA-TOC, absence of efficient standard treatment as assessed by the local multidisciplinary neuroendocrine tumour board, ECOG ≤ 2 and confirmed of SSTR2 expression on [^68^Ga]Ga-DOTA-TOC/-TATE PET/CT. SSTR2 expression was defined by visually increased tracer uptake on [^68^Ga]Ga-DOTA-TATE/[^68^Ga]Ga-DOTA-TOC PET in all evaluable solid tumours and metastases > 1.5 cm. Main exclusion criteria were extensive radiotherapy involving bone marrow over the last three months before inclusion to the study as well as chemotherapy in the last two months before inclusion. Further inclusion and exclusion criteria are listed in the supplemental data. This study was performed in line with the principles of the Declaration of Helsinki. Approval was granted by the Ethics Committee of Northwest and Central Switzerland (16 April 2025/No 2024–02047) and all patients provided signed, written informed consent.

### Procedures

**Fig. 1:**
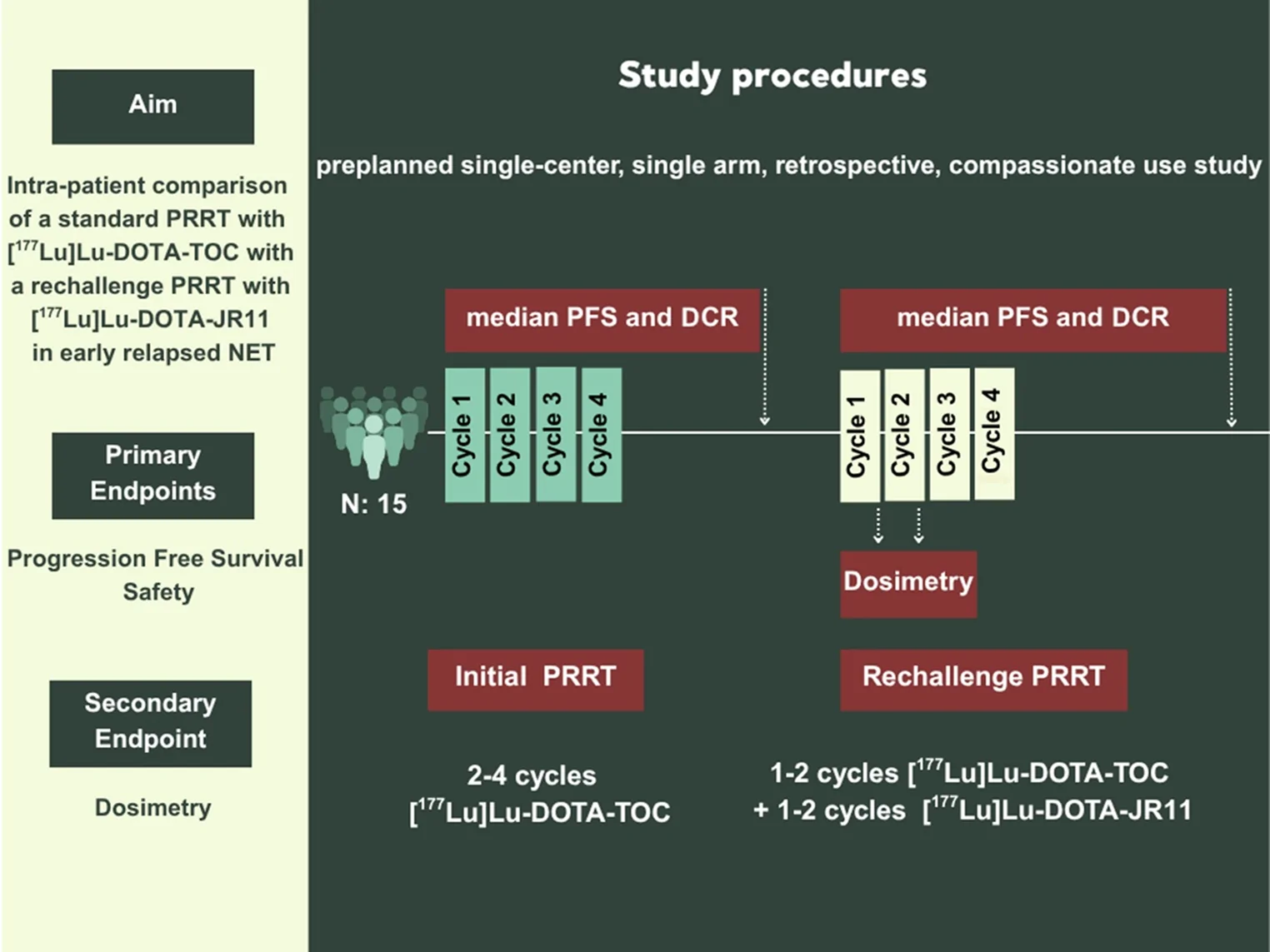
Study procedure overview

Patients with progressive disease ≤ 12 months after PRRT with 2–4 cycles of ~ 7.4 GBq [^177^Lu]Lu-DOTA-TOC were consecutively included and received additional PRRT with 1–2 cycles [^177^Lu]Lu-DOTA-TOC (~ 7.4 GBq) followed by 1–2 cycles [^177^Lu]Lu-DOTA-JR11 (2 GBq/m^2^ x body surface area) at an interval of about 10 weeks. The injected amount of DOTA-JR11 (peptide amount) per cycle was approximately 240 µg, resulting in specific activities of 26–35 GBq/µmol for [^177^Lu]Lu-DOTA-JR11, comparable to those reported in a prospective multicentre phase I/II study (~ 300 µg; ~26 GBq/µmol [^177^Lu]Lu-DOTA-JR11) [12]. Higher peptide amounts (for example 700 µg [^177^Lu]Lu-DOTA-JR11) have been shown to reduce tumour absorbed dose [13]. A body surface area–adapted injected activity (2 GBq/m²) was applied, as fixed activities (for example 3 × 4.5 GBq; ~300 µg [^177^Lu]Lu-DOTA-JR11) may disproportionately increase the risk of clonal hematopoietic stem/progenitor cell or lymphoid neoplasms in women, who typically have a lower body surface area than men [12]. Figure 1 shows an overview about the study procedure, aim and endpoints. To avoid nephrotoxicity 1000 ml of physiologic NaCl solution, which contained 20.0 mg/ml lysine and 20.7 mg/ml arginine, was continuously infused as kidney protection over 5 h. About one hour after the start of this infusion, [^177^Lu]Lu-DOTA-TOC or [^177^Lu]Lu-DOTA-JR11 were infused over one minute or two hours, respectively. Quantitative SPECT/CT scans were performed at ~ 24, ~48 and ~ 168 h after injection of both compounds using a Symbia Intevo 16 system (Siemens Erlangen, Germany) equipped with a medium-energy low-penetration (MELP) collimator.

DOTA-TOC and DOTA-JR11 were synthesized according to good manufacturing practice by Bachem AG (Switzerland) and piChem (Austria), respectively. [^177^Lu]Lu-DOTA-JR11 was manufactured on an automated synthesis module (Pharmtracer, Eckert & Ziegler, Germany). For further information about the preparation of [^177^Lu]Lu-DOTA-TOC and [^177^Lu]Lu-DOTA-JR11 as well as posttreatment SPECT/CT imaging see supplemental data [14].

### Treatment response evaluation

Progression-free survival was defined as the interval from [^177^Lu]Lu-DOTA-TOC therapy start to documented disease progression or death from any cause. Objective tumour assessment by contrast-enhanced [^68^Ga]Ga-DOTA-TOC/-TATE PET/CT, CT and MRI were normally performed at 3 months intervals, and occasionally at 6 months, reflecting the variability inherent to a real-world retrospective study design. Disease progression and response was determined by independent, local, and external experienced bord certified radiologists and nuclear medicine physicians in agreement according to RECIST 1.1 criteria.

### Safety

Vital parameters such as heart frequency, blood pressure and oxygen saturation were collected during the two hour infusion of [^177^Lu]Lu-DOTA-JR11. Blood sampling (a comprehensive metabolic panel as well as a full blood count) was carried out on each therapy day and 2, 4, and 6 weeks thereafter.

Kidney and liver functions (creatinine, ALAT, hypoalbuminemia), haematological disfunctions (anaemia, lymphopenia, neutropenia, thrombocytopenia, or myelodysplasia) were evaluated before therapy and up to 18 months post-therapy. Most common clinical adverse reactions, related to radiopharmaceuticals administration were assessed: abdominal pain, nausea, flush, diarrhoea, constipation, hypotension, vomiting or other neoplasia’s. Only worsening of symptoms after start of [^177^Lu]Lu-DOTA-TOC or [^177^Lu]Lu-DOTA-JR11 therapy was graded according to the Common Terminology Criteria for Adverse Events (CTCAE) v5.0. All adverse reactions were assessed at least 5 months and up to 18 months posttherapy.

### Dosimetry

The volume of tumours was determined by manual segmentation of native CT images acquired from post-therapy SPECT/CT scans. Volumetry of kidneys and spleen was based on an automated AI segmentation tool. The uptake of lesions and organs was derived from VOIs on the NM dataset. The number of disintegrations and the absorbed doses were calculated with OLINDA 1.0. The phantom organ weight was adjusted to the patient organ weight for kidneys, liver and spleen. The tumour absorbed dose was calculated using the spheres model in OLINDA.

The red marrow activity was determined by delineating volumes of interest (VOIs) in each vertebra from Th2 to L5 at all imaging time points. The absorbed dose to red marrow was then calculated by multiplying the time-integrated activity concentration by the absorbed energy per decay of ^177^Lu. For additional methodological details, see the supplemental data.

### Statistical analysis

All data were summarized using IBM SPSS Statistics for Windows, Version 29.0.2.0 (Released 2022; IBM Corp, Armonk, NY: USA). Unless otherwise stated, data are expressed as median with range. We used Chi quadrat and Wilcoxon test, as appropriate, to compare proportions or median differences. Two-sided p value was considered significant if < 0.05. Kaplan-Meier survival analysis curves were generated to assess and compare progression free survival after initiation of [^177^Lu]Lu-DOTA-TOC alone and after initiation of [^177^Lu]Lu-DOTA-TOC + [^177^Lu]Lu-DOTA-JR11 therapy. The endpoint was defined as progression or death and was assigned a value of 1 if achieved and 0 if not achieved.

Stacked bar Chart were created with IBM SPSS to compare progression free survival after [^177^Lu]Lu-DOTA-TOC and after [^177^Lu]Lu-DOTA-TOC + [^177^Lu]Lu-DOTA-JR11 therapy for each patient of the study population. Patient numbers are given in chronological order.

## Results

**Fig. 2:**
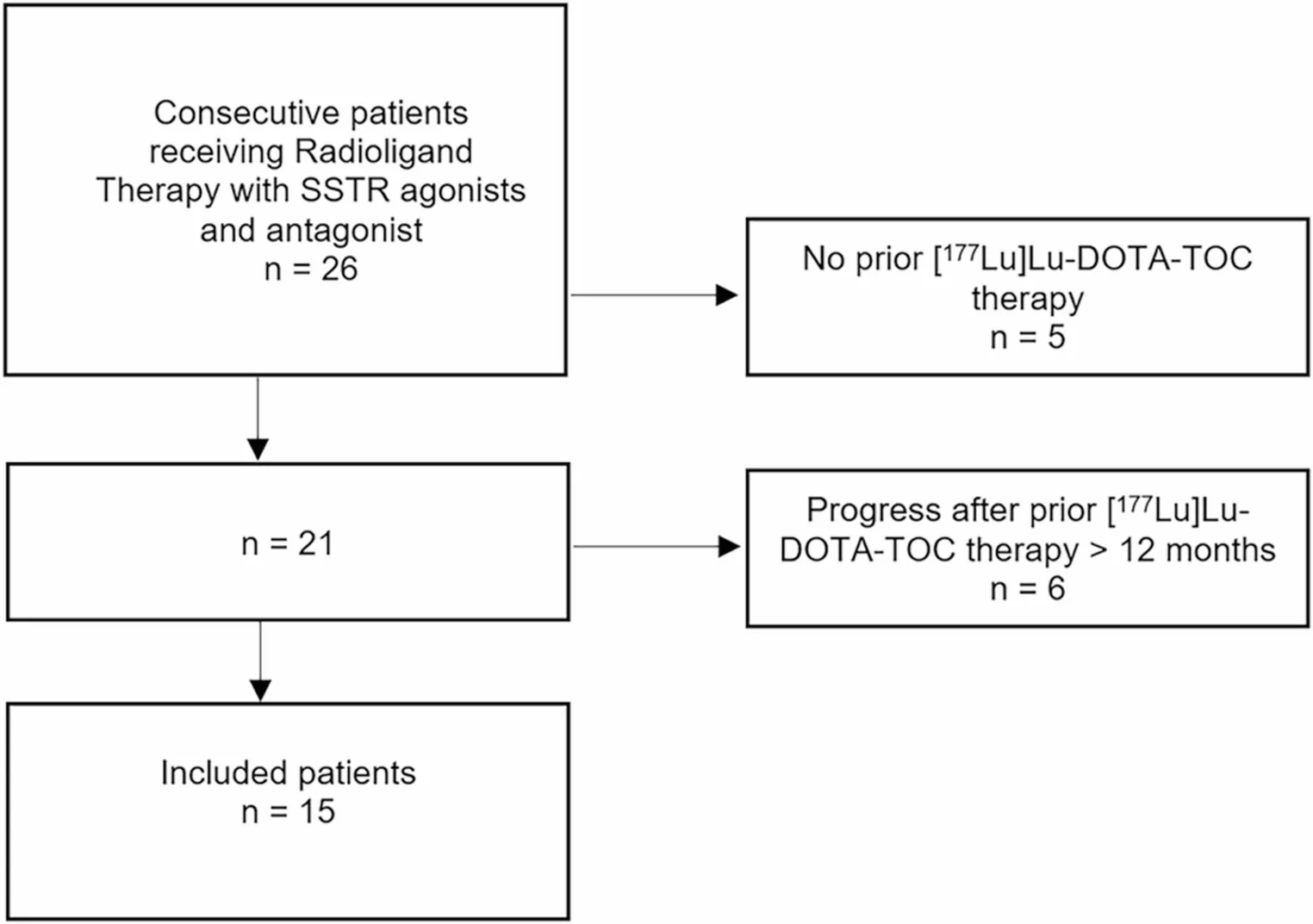
Flowchart of patient selection

From April 2021 until May 2023, 15 patients with a median age of 56.2 years (range: 25–76) were included. Figure 2 shows the flow chart of the study. The cohort consisted of 12 males and 3 females. The median time since initial diagnosis was 57 months (range: 24–139), and the median time from last PRRT to disease progression was 9 months (range: 1–12). The most common primary tumour types were gastrointestinal (60%) and pancreatic (20%). Functioning tumours were observed in 60% of patients. For further patient characteristics see Table 1.

**Table 1:** Summary of patient characteristics, treatment protocol, remission status and adverse events

Characteristics	All Patients (*N* = 15)
Age (years)	
Median (range)	56.2 (25–76)
Sex	
Male	12 (80%)
Female	3 (20%)
Time since initial diagnosis (months)	
Median (range)	57 (24–139)
Mean ± SD	75.2 ± 39.18
Time from last PRRT to progress (months)	
Median (range)	9 (1–12)
Mean ± SD	7.2 ± 4.0
Primary tumour type	
Gastrointestinal	9 (60%)
Pancreatic	3 (20%)
Lung	1 (7%)
Paraganglioma or pheochromocytoma	1 (7%)
Thymus	1 (7%)
Unknown	0 (0%)
Site of Metastasis	
Lymph nodes	12 (80%)
Liver	12 (80%)
Bone	11 (73%)
Peritoneum	4 (27%)
Lungs	3 (20%)
Orbit	1 (7%)
Pancreas	1 (7%)
Hedinger-Syndrome	4 (27%)
With tricuspid valve replacement	4 (27%)
Without tricuspid valve replacement	0 (0%)
Neuroendocrine Tumour Grade (WHO)	
1	4 (27%)
2	8 (53%)
3	3 (20%)
Net functionality	
Functioning	9 (60%)
Non-functioning	6 (40%)
Prior treatments	
Somatostatin Analogues	13 (87%)
PRRT	15 (100%)
Surgery	11 (73%)
Chemotherapy	4 (27%)
Radiotherapy	3 (20%)
RFA	1 (7%)
TACE	1 (7%)
TKI	1 (7%)
SIRT	1 (7%)

*Abbreviations*: *PRRT *Peptide receptor radionuclide therapy, *RFA* Radiofrequency ablation, *TACE* Transarterial chemoembolization, *TKI* Tyrosine kinase inhibitor, *SIRT* Selective internal radiation therapy

Data are reported as *n* (%) unless stated otherwise

All patients had previously received standard PRRT with [^177^Lu]Lu-DOTA-TOC (2–4 cycles, 5.6–7.5 GBq/cycle; 39 cycles in total, mean cumulative number of 2.5 cycles per patient). Upon progression, patients received a rechallenge PRRT with 1–2 additional cycles of ~ 190 µg [^177^Lu]Lu-DOTA-TOC (5.1–7.4 GBq/cycle; 18 cycles in total, mean cumulative number of 1.2 cycles per patient), followed by 1–2 cycles of ~ 240 µg [^177^Lu]Lu-DOTA-JR11 (2.8–4.8 GBq/cycle; 18 cycles in total, mean cumulative number of 1.2 cycles per patient), administered at intervals of 10 ± 1 weeks. The variability in the administered activity of [^177^Lu]Lu-DOTA-TOC was primarily determined by suboptimal blood parameters. Table 2 provides an overview of the number of cycles in the initial/standard and rechallenge PRRT groups, demonstrating a balanced distribution of cycles between initial/previous and rechallenge PRRT, which was considered a key prerequisite for comparing PFS within the same patients.

**Table 2:** Treatment distribution across treatment phase

Parameter	Treatment phase		
Number of cycles received by treatment phase	Initial/previous PRRT	Rechallenge PRRT	
	[^177^Lu]Lu-DOTA-TOC (*N* = 15)	[^177^Lu]Lu-DOTA-TOC (*N* = 15)	[^177^Lu]Lu-DOTA-JR11 (*N* = 15)
1	0 (0%)	12 (80%)	12 (80%)
2	10 (67%)*	3 (20%)	3 (20%)
3	2 (13%)	0 (0%)	0 (0%)
4	3 (20%)	0 (0%)	0 (0%)
Cumulative number of cycles	39	18	18
	Initial/previous PRRT	Rechallenge PRRT	
Mean cumulative number of cycles per patient	2.5 ± 0.8	1.2 ± 0.4	1.2 ± 0.4
		2.4 ± 0.8	
Cumulative administered activity (GBq)	273	125	69
Total cumulative administered activity (GBq)	273	194	

Qualitative data are number and percentage; continuous data are mean ± SD. *In the initial/previous PRRT phase, 10 patients were not PRRT-naïve and therefore received only two cycles of [^177^Lu]Lu-DOTA-TOC

### Treatment response

**Fig. 3:**
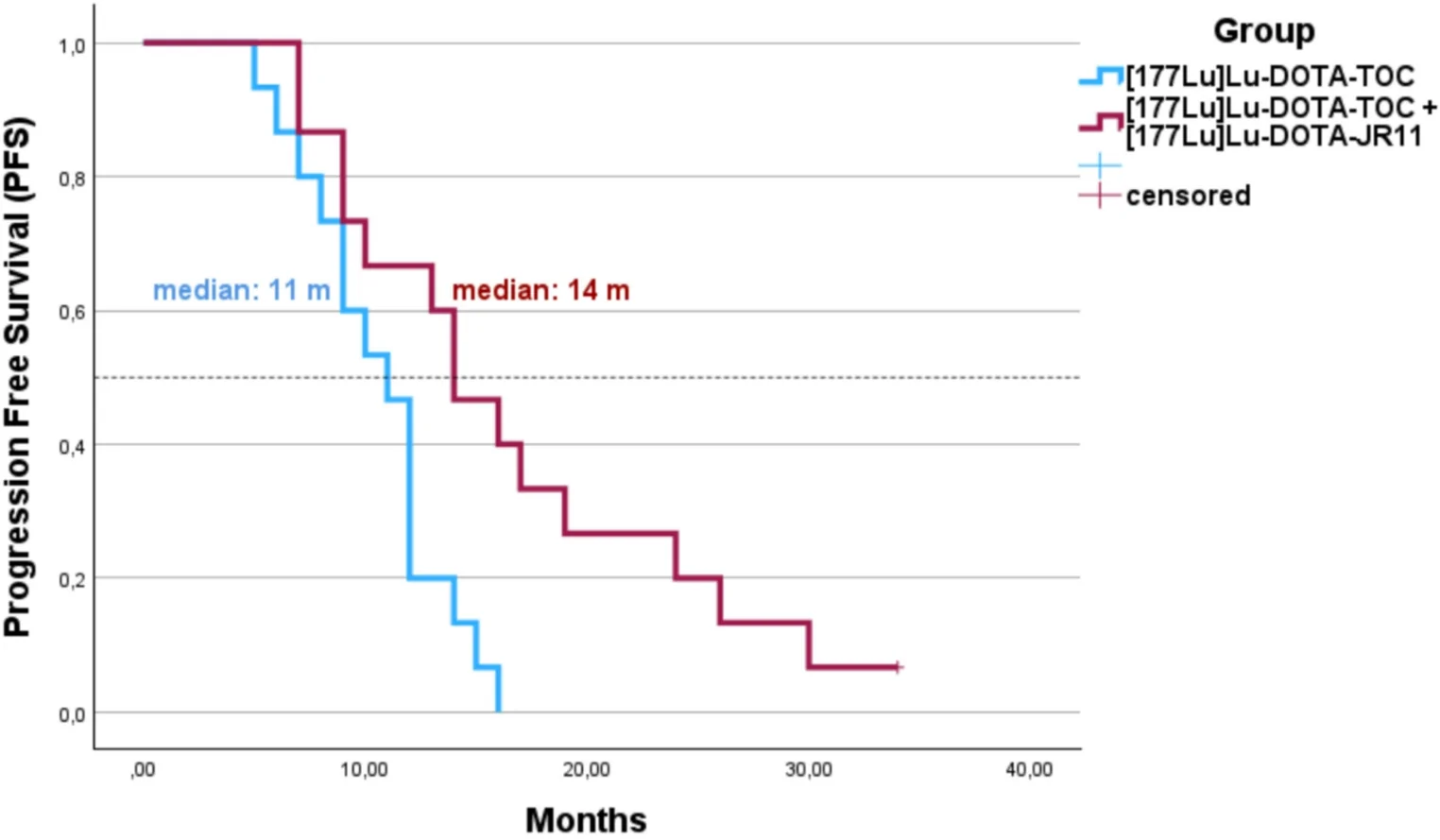
Kaplan Meier analysis of progression free survival as assessed by regular follow-up imaging after therapy with 2–4 cycles [^177^Lu]Lu-DOTA-TOC until progression (blue line, total of 39 cycles [^177^Lu]Lu-DOTA-TOC). The red line shows rechallenge therapy with 1–2 cycles [^177^Lu]Lu-DOTA-TOC therapy (total of 18 cycles) + 1–2 cycles [^177^Lu]Lu-DOTA-JR11 therapy (total of 18 cycles) in the same patients. Hazard ratio ~ 0.34 (95%CI 0.14-0,81; log-rank p: 0.007)

Figure 3 presents a Kaplan–Meier analysis of progression-free survival (PFS), determined by regular follow-up imaging. PFS was compared after 2–4 cycles of [^177^Lu]Lu-DOTA-TOC versus 1–2 cycles of [^177^Lu]Lu-DOTA-TOC followed by 1–2 cycles of [^177^Lu]Lu-DOTA-JR11 in the same patients. The median PFS following standard PRRT with [^177^Lu]Lu-DOTA-TOC was 11 months (95% CI, 9–13), whereas treatment with [^177^Lu]Lu-DOTA-TOC plus [^177^Lu]Lu-DOTA-JR11 yielded a median PFS of 14 months (95% CI, 10–18). The hazard ratio for disease progression or death, comparing rechallenge PRRT with [^177^Lu]Lu-DOTA-TOC plus [^177^Lu]Lu-DOTA-JR11 to the standard PRRT, was ~ 0.34 (*P* = 0.007). In our patient cohort the DCR was 20% for standard PRRT and 67% for rechallenge PRRT with [^177^Lu]Lu-DOTA-TOC plus [^177^Lu]Lu-DOTA-JR11 12 months after start of therapy.

**Fig. 4:**
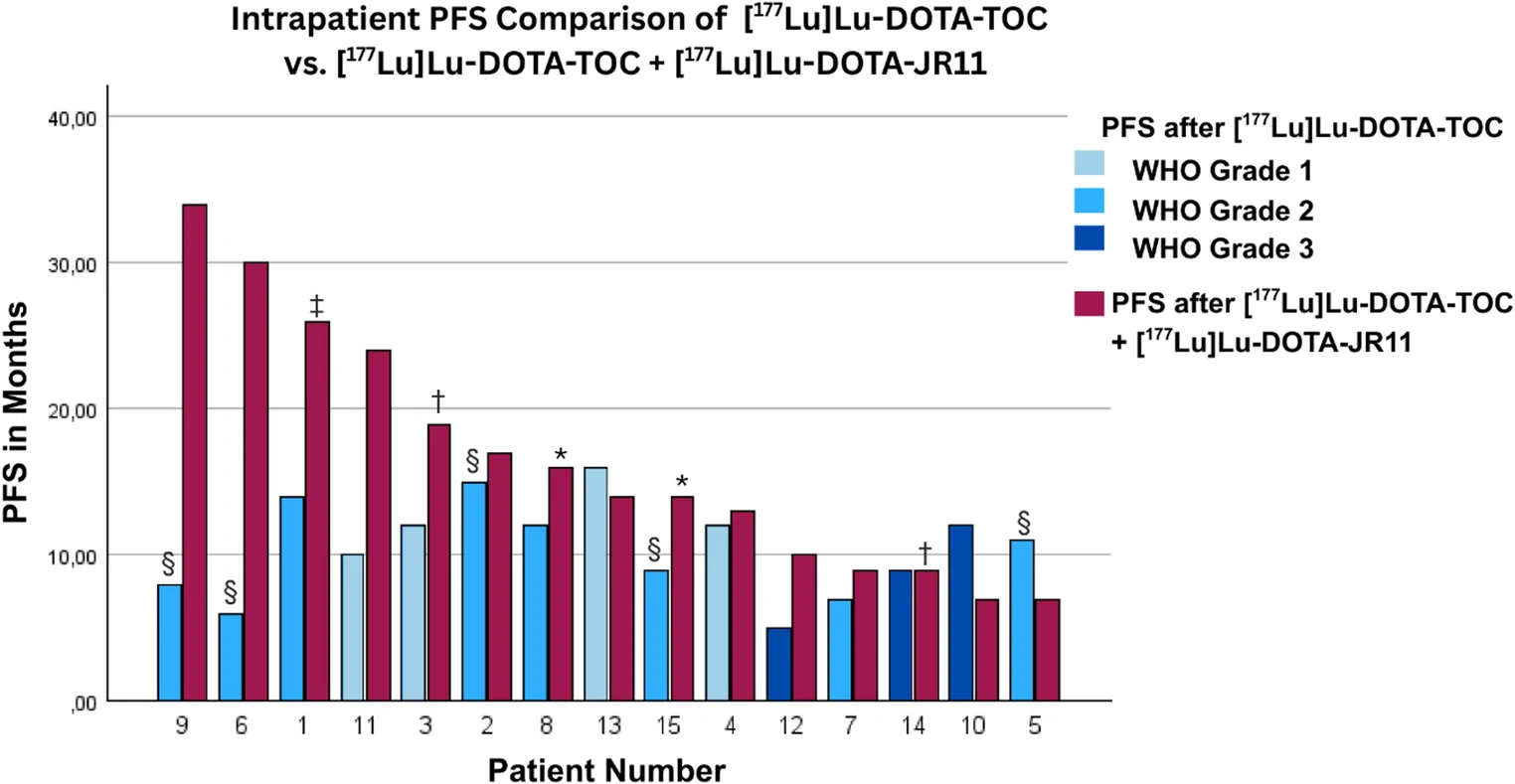
Stacked bar chart showing comparison of progression free survival and WHO tumour grade for each patient following initial/previous PRRT and subsequent rechallenge PRRT. Blue shading indicates WHO tumor grade, with light blue representing grade 1 (G1), medium blue grade 2 (G2), and dark blue grade 3 (G3). Most patients were not PRRT-naïve and therefore received only 2 cycles [^177^Lu]Lu-DOTA-TOC as initial/previous PRRT (blue) before undergoing rechallenge PRRT with one cycle of [^177^Lu]Lu-DOTA-TOC and one cycle of [^177^Lu]Lu-DOTA-JR11 (violet). Exceptions: *2 patients received 2 x [^177^Lu]Lu-DOTA-TOC + 1 x [^177^Lu]Lu-DOTA-JR11 as rechallenge PRRT. ^†^2 patients received 1 x [^177^Lu]Lu-DOTA-TOC + 2 x [^177^Lu]Lu-DOTA-JR11 as rechallenge PRRT. ^‡^1 patient received 2 x [^177^Lu]Lu-DOTA-TOC + 2 x [^177^Lu]Lu-DOTA-JR11 as rechallenge PRRT. ^§^5 initially PRRT-naïve patients received 3–4 x [177Lu]Lu-DOTA-TOC as initial/previous PRRT. Patients 3, 6, 9, 11, and 15 were followed at approximately 6-month intervals, reflecting an indolent disease course and/or low WHO grade. All other patients were assessed at approximately 3-month intervals, consistent with a more aggressive disease course and/or higher tumor WHO grade

Figure 4 provides a per-patient comparison of PFS for both therapy regimens. Patients who underwent any deviation from the standard protocol were explicitly marked. Three of the 15 patients achieved slightly longer PFS with [^177^Lu]Lu-DOTA-TOC alone, whereas 11 experienced longer PFS after rechallenge PRRT with [^177^Lu]Lu-DOTA-TOC plus [^177^Lu]Lu-DOTA-JR11. Response assessment according to RECIST 1.1 demonstrated stable disease as the best objective response following PRRT rechallenge in all patients. 

**Fig. 5:**
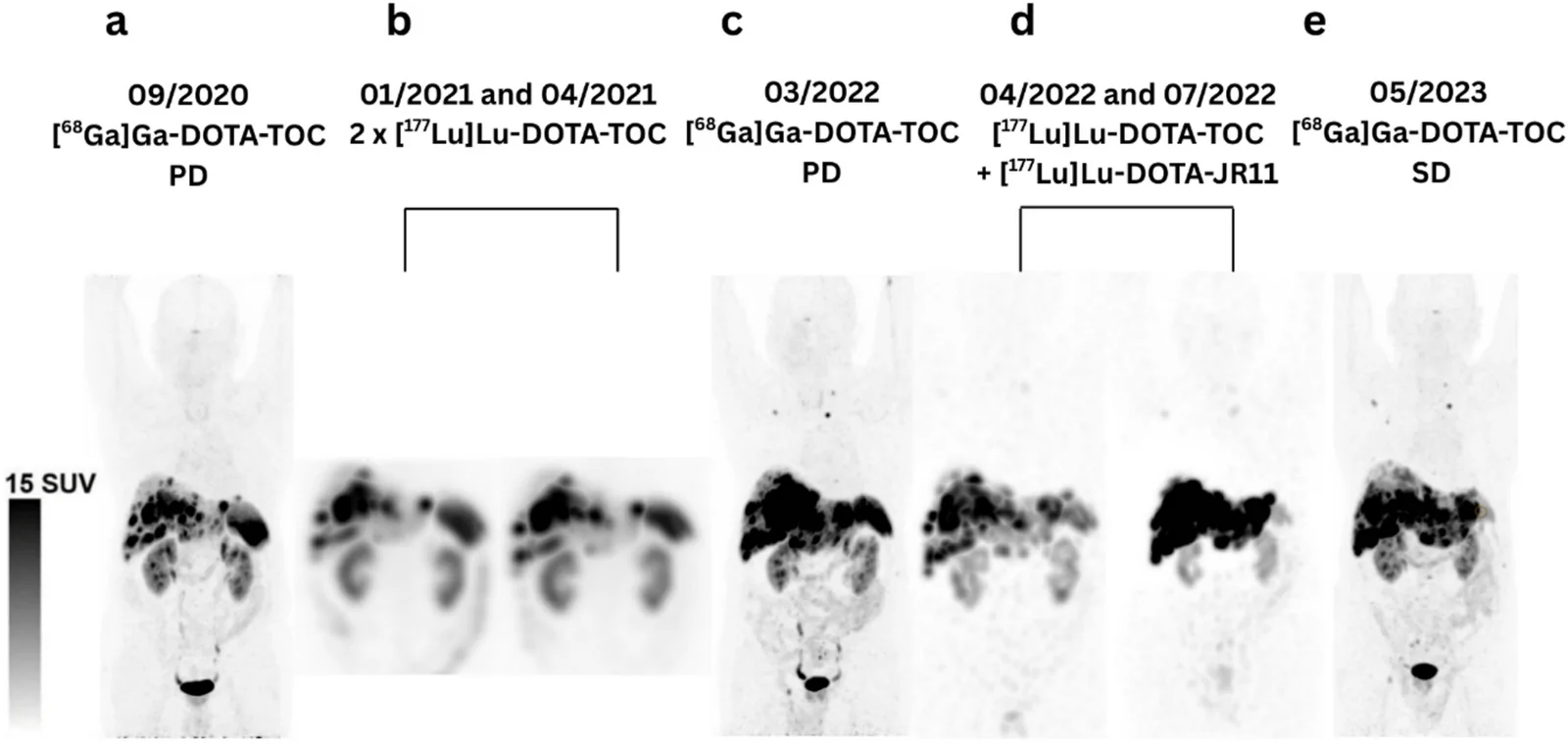
Maximum intensity projections of [⁶⁸Ga]Ga-DOTA-TOC PET/CT and post-therapeutic SPECT/CT in patient 11. **a **[⁶⁸Ga]Ga-DOTA-TOC PET/CT (09/2020) demonstrating progressive disease (PD). **b** Post-therapeutic SPECT/CT after two cycles of [¹⁷⁷Lu]Lu-DOTA-TOC administered in 01/2021 and 04/2021. **c** [⁶⁸Ga]Ga-DOTA-TOC PET/CT (03/2022) demonstrating progressive disease with progressive liver and new bone metastases. **d** Post-therapeutic SPECT/CT after PRRT re-challenge: one cycle [¹⁷⁷Lu]Lu-DOTA-TOC (04/2022) and one cycle [¹⁷⁷Lu]Lu-DOTA-JR11 (07/2022). **e **[⁶⁸Ga]Ga-DOTA-TOC PET/CT (05/2023) demonstrating stable disease (SD). The first follow-up after the initial PRRT was performed using MRI

Supplemental Fig. 1 compares (in all 15 patients) post-treatment maximum intensity projection (MIP) images obtained by quantitative SPECT 48 h after injection of [^177^Lu]Lu-DOTA-TOC and after [^177^Lu]Lu-DOTA-JR11 in the same patients, imaged 10 ± 2 weeks apart. In nearly all cases, tumour uptake was markedly higher with [^177^Lu]Lu-DOTA-JR11. Figure 5 illustrates the clinical course of the disease in patient 8.

### Safety

All adverse events are summarized in Table 3. No hypotension or vomiting was noted after injection of both compounds. There were two grade 3 adverse events (lymphopenia, hypoalbuminemia) after therapy with [177Lu]Lu-DOTA-TOC, and six grade 3 adverse events (anaemia, lymphopenia) after therapy with [177Lu]Lu-DOTA-JR11 (Table 3). In all patients the reported adverse events resolved after few weeks. No grade 4 or 5 adverse events were observed. Up to 18 Months after the first study cycle there was no worsening of kidney function or new evidence for neoplasms (e.g. myelodysplastic syndrome).

**Table 3:** Adverse events of rechallenge PRRT during 5–18 months of follow-up

	[^177^Lu]Lu-DOTA-TOC(N = 15)		[^177^Lu]Lu-DOTA-JR11(N = 15)	
	Number of patients (percent)			
Adverse event	Grade 1–3	Grade 3	Grade 1–3	Grade 3
Any	12 (80%)	2 (13%)	13 (87%)	6 (40%)
Related to therapy	10 (67%)	1 (7%)	13 (87%)	6 (40%)
Anaemia	2 (13%)	0 (0%)	8 (53%)	3 (20%)†
Thrombocytopenia	2 (13%)	0 (0%)	8 (53%)	0 (0%)
Lymphopenia	5 (33%)	1 (7%)*	9 (60%)	4 (27%)‡
Neutropenia	1 (7%)	0 (0%)	1 (7%)	0 (0%)
Hypoalbuminemia	3 (20%)	1 (7%)*	3 (20%)	0 (0%)
ALAT	5 (33%)	0 (0%)	3 (20%)	0 (0%)
Creatinine	0 (0%)	0 (0%)	2 (13%)	0 (0%)
Abdominal pain	2 (13%)		1 (7%)	
Nausea	1 (7%)		1 (7%)	
Flush	0 (0%)		1 (7%)	
Diarrhoea	0 (0%)		1 (7%)	
Constipation	0 (0%)		1 (7%)	
Vomiting	0 (0%)		0 (0%)	
Hypotension	0 (0%)		0 (0%)	
Myelodysplastic Syndrome or other Neoplasia	0 (0%)		0 (0%)	

There was no grade 4 and 5 toxicity. All Grade 3 events were reversible

* Patient 12 and patient 8 received each 1 cycle of [^177^Lu]Lu-DOTA-TOC

† Patients 5, 6, and 11 received each 1 cycle of [^177^Lu]Lu-DOTA-JR11

‡ Patients 2, 5, 12 and 15 received each 1 cycle of [^177^Lu]Lu-DOTA-JR11

### Dosimetry

Table 4 shows the results of tumour absorbed-dose estimations and tumour-to-kidney dose ratios for all 15 patients. Red marrow dose could not be calculated for 9 patients due to disseminated bone metastases in the spine (7 patients) or image reconstruction issues (2 patients).

**Table 4:** Summary of tumour radiation dose estimations

Patient	[^177^Lu]Lu-DOTA-TOC*				[^177^Lu]Lu-DOTA-JR11^†^				[^177^Lu]Lu-DOTA-JR11/ [^177^Lu]Lu-DOTA-TOC
	Tumour	Red marrow			Tumour	Red marrow			
No.	Dose^‡^	Dose^§^	T/BM^§^	T/K	Dose^‡^	Dose^§^	T/BM^§^	T/K	
	(Gy/cycle)	(Gy/cycle)	Ratio	Ratio	(Gy/cycle)	(Gy/cycle)	Ratio	Ratio	Tumour dose ratio
1	4.8			5.7	11.7			10.8	2.4
2	0.8	0.21	4	0.2	1.0	0.65	2	0.2	1.2
3	30.2	0.41	74	8.0	37.2	0.54	69	12.2	1.2
4	34.0			17.2	167			91.1	4.9
5	1.3	0.20	6	0.5	1.1	0.54	2	0.2	0.9
6	3.9	0.23	17	0.9	24.5	0.66	37	3.7	6.4
7	18.9			15.8	60.2			29.7	3.2
8	34.1			17.0	86.6			54.5	2.5
9	4.2	0.15	28	2.2	15.1	0.32	47	11.2	3.6
10	19.0			10.2	25.7			13.5	1.4
11	223			109	353			265	1.6
12	4.2	0.11	38	2.2	8.9	0.37	24	2.7	2.1
13	12.9			5.3	17.1			6.1	1.3
14	8.0			2.1	18.5			4.0	2.3
15	13.9			7.8	14.0			6.3	1.0
Median	12.9	0.21	22	5.7	18.5	0.54	31	10.8	2.1
(range)	0.8–223	0.11–0.41	4–74	0.2–109	1.0-354	0.32–0.66	2–69	0.2–265	0.9–6.4

*[^177^Lu]Lu-DOTA-TOC therapy cycles were performed with a medium activity of 7.3 GBq (range: 5.1–7.4)

^†^[^177^Lu]Lu-DOTA-JR11 therapy cycles were conducted with 2 GBq x body surface area +/-10% per cycle

^‡^The tumour dose of both compounds is given as the mean of the same 1–3 tumours per patient

^§^Red marrow dose could not be calculated for 9 patients due to bone metastases in the spine or image reconstruction issues (2 patients)

*T/BM* Tumour-to-red bone marrow dose ratio, *T/K* Tumour-to-kidney dose ratio

The median of the mean tumour dose was 2.1 (range, 0.9–6.4) times higher with [^177^Lu]Lu-DOTA-JR11 compared to [^177^Lu]Lu-DOTA-TOC. Tumour-to-kidney and tumour-to-spleen dose ratios were also favourable with [^177^Lu]Lu-DOTA-JR11 being 1.9 (range, 0.4–5.3) and 2.7 (range, 1.2-6.0) times higher, respectively. The median of the tumour–to–bone marrow dose ratio of 6 patients was similar for both compounds. Absorbed-dose estimations for most relevant organs with [^177^Lu]Lu-DOTA-TOC and [^177^Lu]Lu- DOTA-JR11 are summarized in supplemental Table 1.

## Discussion

This single arm therapy study compares for the first time median PFS and DCR of a SSTR2 agonist (standard PRRT with [^177^Lu]Lu-DOTA-TOC) with an antagonist (rechallenge PRRT with [^177^Lu]Lu-DOTA-JR11) in the same patient with a SSTR2 positive NET.

The main results of this study can be summarized as follows: First, median PFS was 3 months longer after rechallenge PRRT with [^177^Lu]Lu-DOTA-TOC plus [^177^Lu]Lu-DOTA-JR11 than after standard PRRT with [^177^Lu]Lu-DOTA-TOC alone (Hazard ratio of ~ 0.34) resulting in a disease control rate of 67% versus 20% twelve months after therapy start. Despite a lower total cumulative administered activity during the rechallenge PRRT (194 GBq) compared to the initial/previous PRRT (273 GBq), improved clinical outcomes were observed. This finding suggests that the enhanced efficacy may be associated with the inclusion of [¹⁷⁷Lu]Lu-DOTA-JR11, rather than to the cumulative activity. The superiority of [^177^Lu]Lu-DOTA-JR11 can be explained by a median tumour absorbed dose which is about twice as high per therapy cycle than with [^177^Lu]Lu-DOTA-TOC although [^177^Lu]Lu-DOTA-JR11 therapy was performed with 1.4–2.5 times lower activities. The peptide amount, specific activity (GBq/µmol), molar activity and number of therapy cycles were in the same range for both compounds and across treatment phases, making them unlikely contributors to the improved performance of [¹⁷⁷Lu]Lu-DOTA-JR11. A lower molar activity of the radiopharmaceutical might be of relevance as a lower molar activity can lead to a saturation effect on SSTR2 positive tumour cells reducing the absorbed dose to the tumour [13, 15].

Second, the median tumour (range) absorbed dose of [^177^Lu]Lu-DOTA-JR11 was high with 19 Gy (1.0–354 Gy) per cycle and was higher than with [^177^Lu]Lu-DOTA-TOC in 28 of 30 tumours.

Third, retreatment with 1–2 cycles [^177^Lu]Lu-DOTA-TOC (standard injected activity) plus 1–2 cycles [^177^Lu]Lu-DOTA-JR11 (2 GBq/m^2^ x body surface area) was safe with reversible grade 3 toxicity, no grade ≥ 4 toxicity and no induction of therapy-related myeloid neoplasms up to 18 months after therapy start in this heavily pretreated patient collective. Renal toxicity is expected to be negligible with [^177^Lu]Lu-DOTA-JR11 due to the low median (range) absorbed kidney dose of 2.22 (1.08–6.67) Gy/cycle and a median tumour-to-kidney absorbed dose ratio per cycle which is two times higher than with [^177^Lu]Lu-DOTA-TOC. Tumour-to-bone marrow dose ratios of both compounds were in the same range although estimated absorbed bone marrow dose was 1.3–3.4 times higher per therapy cycle with [^177^Lu]Lu-DOTA-JR11. Lastly, the low hematologic toxicity after rechallenge PRRT with 1–2 cycles [^177^Lu]Lu-DOTA-JR11 justifies more rechallenge cycles in future studies.

The results of this compassionate use, single centre study give rise to the expectation that [^177^Lu]Lu-DOTA-JR11 therapy results in a better tumour control rate than PRRT with [^177^Lu]Lu-DOTA-TOC in patients with advanced metastatic NETs. On the other hand, bone marrow toxicity seems to be a limitation for the clinical use of radiolabelled SSTR2 antagonists. In clinical studies, higher numbers of grade ≥ 3 hematologic toxicity were shown in patients treated with SSTR2 antagonists like [^177^Lu]Lu-DOTA-JR11 or [^177^Lu]Lu-DOTA-LM3 [10–12] compared to patients treated with SSTR2 agonists like [^177^Lu]Lu-DOTA-TOC or [^177^Lu]Lu-DOTA-TATE [5, 16, 17]. Previous studies have demonstrated that human haematopoietic cells, particularly CD34 + progenitor cells, express SSTR2 [18, 19]. This may explain the enhanced haematotoxicity observed with [^177^Lu]Lu-DOTA-JR11, as [^177^Lu]Lu-DOTA-JR11 exhibit a higher ability to bind to SSTR2-expressing hematopoietic stem and progenitor cells compared to [^177^Lu]Lu-DOTA-TOC [19]. However, haematotoxicity was low in our study compared to previously published results [10, 12]. There are at least two possible reasons which may explain the lower haematotoxicity in our study: First, determination of treatment (injected) activity in our study was based on size and weight of patients (2 GBq/m^2^ x body surface area). With this personalized therapy approach the median (range) injected activity of [^177^Lu]Lu-DOTA-JR11 was lower (3.8 (2.8–4.8) GBq/cycle) compared to studies with standard injected activities (~ 7 and ~ 4.5 GBq/cycle) [10, 12]. The lower injected activity results in a lower absorbed dose to the bone marrow and subsequently less bone marrow toxicity as there is a direct correlation between injected activity and bone marrow dose. Second, the peptide amount of [^177^Lu]Lu-DOTA-JR11 was in our study several times higher than in the study of Reidy-Lagunes et al. (~ 240 µg vs. <100 µg) [10]. This may reduce the absorbed dose to the bone marrow as a higher peptide amount (= lower molar activity) of the radiopharmaceutical can have a saturation effect on SSTR2 positive cells in the bone marrow [14, 15].

This study has limitations. First, the relatively small sample size may limit the generalizability of the findings. Although the hazard ratio for progression-free survival was significant, the result should be interpreted cautiously and confirmed in larger studies. Second, this is a compassionate use study with a personalized approach based on a multidisciplinary tumour board recommendation. Therefore, the study reflects a real-world approach with a personalized number of rechallenge therapy cycles. Ten patients were not PRRT-naïve and therefore received only 2 cycles [177Lu]Lu-DOTA-TOC as their initial/previous PRRT before undergoing rechallenge PRRT. In line with standard practice at our centre and others, non-PRRT-naïve patients are typically treated with two PRRT cycles [6, 20]. Third, tumor reassessment intervals were not uniform across all patients. Patients with an indolent disease course (WHO grade 1–2) were typically followed at around 6-month intervals, whereas patients with more aggressive disease behaviour and/or higher tumour grade were assessed at around 3-month intervals. This difference in follow-up frequency, although reflecting a real-world clinical approach, may have influenced the timing of progression detection and, consequently, progression-free survival (PFS) estimates and disease control rate (DCR) comparisons. Fourth, bone marrow dosimetry data were available for only 6 of 15 patients. In 7 patients, dosimetry could not be performed due to disseminated bone metastases, and in 2 patients because of reconstruction issues. This limited number of evaluable cases restricts the robustness of the bone marrow dosimetry analysis. Fifth, during rechallenge PRRT, the sequential administration of therapeutic doses of [^177^Lu]Lu-DOTA-TOC followed ~ 10 weeks later by [^177^Lu]Lu-DOTA-JR11 may lead to carry-over effects, affecting both toxicity and therapeutic efficacy, from the treatment with [^177^Lu]Lu-DOTA-TOC onto the treatment with [^177^Lu]Lu-DOTA-JR11. Thus, measurement of [^177^Lu]Lu-DOTA-JR11 toxicity could impose falsely higher numbers and grade of adverse events. Furthermore, dosimetry calculation could reflect a falsely lower tumor absorbed dose estimation of [^177^Lu]Lu-DOTA-JR11 because of the carry-over effect.

## Conclusion

The median tumour absorbed dose per cycle was approximately twofold higher with [^177^Lu]Lu-DOTA-JR11 than with standard [^177^Lu]Lu-DOTA-TOC therapy in the same patients, resulting in a median PFS benefit of 3 months in our cohort of early-progressing NET patients following standard PRRT. The encouraging preliminary results of higher efficacy and low hematologic toxicity of [^177^Lu]Lu-DOTA-JR11 in our compassionate use study warrant further evaluation of [^177^Lu]Lu-DOTA-JR11 in larger, prospective phase II or III studies.

## Supplementary Information

Supplementary Material 1.

## Data Availability

The datasets generated during and/or analysed during the current study are available from the corresponding author on reasonable request.
