# Supplementary material for: Increased progression free survival with radiolabelled somatostatin receptor antagonist versus standard peptide receptor radionuclide therapy in patients with somatostatin receptor positive tumours
Source: Eur J Nucl Med Mol Imaging. 2026 Mar 24;53(9):5219–29. doi: 10.1007/s00259-026-07856-9 (PMC13652862; doi:10.1007/s00259-026-07856-9)
Supplement: Supplementary file 1 [file 259_2026_7856_MOESM1_ESM.docx]

**Supplemental Data**

**Increased Progression Free Survival with Radiolabelled Somatostatin Receptor Antagonist versus standard Peptide Receptor Radionuclide Therapy in Patients with Somatostatin Receptor Positive Tumours**

**Supplemental materials and methods**

Inclusion criteria:

1) Written informed consent.

2) Patient of any gender and of age older than 18 years.

3) Female patients capable of giving birth (who are not surgically sterilized or are less than 2 years in their menopause) must use a medically accepted contraceptive and must agree to use it during and till 3 months after the treatment. As acceptable contraceptive count sexual abstinence or double contraceptive methods: hormonal contraceptive (oral, transdermal, implants or injections) in combination with barrier methods (spiral, condom, diaphragm).

4) Male patients must use medically accepted contraceptive during and till 3 months after treatment.

5) The participants must be patients with histologically confirmed neuroendocrine tumour grade 1-3 with progression ≤ 12 months after termination of standard PRRT with [^177^Lu]Lu-DOTA-TOC.

6) There must be no other standard therapeutic alternatives for the participants (assessment by local multidisciplinary neuroendocrinal tumour board).

7) The ECOG performance status must be ≤ 2.

8) At least one tumour must be measurable in 3 dimensions.

9) The participants must have a confirmed expression of somatostatin receptor subtype 2 (SST2) on ^68^Ga-DOTA-TOC/-TATE PET/CT scan. SSTR2 expression was defined by visually increased tracer uptake on [^68^Ga]Ga-DOTA-TATE/[^68^Ga]Ga-DOTA-TOC PET in all evaluable solid tumours and metastases >1.5 cm.

10) Blood parameters:

a) Leucocytes ≥ 3*10^9^/L

b) Haemoglobin ≥ 80 g/L

c) Thrombocytes ≥ 90*10^9^/L

d) Calculated glomerular filtration rate ≥ 50 mL/min

e) Albumin > 25g/L

f) ALT, AST, AP: ≤ 5 times upper standard value

g) Bilirubin ≤ 2 times upper standard value

Exclusion criteria:

1) Known intolerance against ^177^Lu, DOTA, JR11, TOC or against one of the components of [^177^Lu]Lu-DOTA-JR11 or [^177^Lu]Lu-DOTA-TOC.

2) Ongoing infection at the screening visit or a serious infection in the past 4 weeks.

3) Administration of another investigational product 30 days before and during the ongoing study.

4) Prior or planed administration of a therapeutic radio-pharmaceutical during 8 half-lives of the used radio-pharmaceutical’s radionuclide, also during the ongoing study.

5) Any extensive Radiotherapy involving bone marrow over the last 3 months before inclusion to the study.

6) Chemotherapy in the last 2 months before inclusion.

7) Pregnant or breastfeeding female patients.

8) Any uncontrolled significant medical, psychiatric or surgical condition (active infection, unstable angina pectoris, cardiac arrhythmia, poorly controlled hypertension, poorly controlled diabetes mellitus [HbA1c ≥ 9%], uncontrolled congestive heart disease, etc.) or laboratory findings that might jeopardize the patient's safety or that would limit compliance with the objectives and assessments of the study. Anymental conditions which prevent the patient from understanding the type, extent and possible consequences of the study and/or an uncooperative attitude from the patient.

Preparation of [^177^Lu]Lu-DOTA-TOC and [^177^Lu]Lu-DOTA-JR11:

DOTA-TOC and DOTA-JR11 (1) were synthesized according to good manufacturing practice by Bachem AG (Switzerland) and piChem (Austria), respectively. [^177^Lu]Lu-DOTA-JR11 was manufactured on an automated synthesis module (Pharmtracer, Eckert & Ziegler, Germany): 300 µg DOTA-JR11 were dissolved in sodium acetate/ascorbic acid buffer (pH 4.5) and reacted with 3 - 6 GBq no-carrier-added [^177^Lu]LuCl3 (EndolucinBeta, ITM, Germany) at 83°C for 20 min, followed by C18 solid phase extraction. The final product was formulated in physiological saline solution containing ascorbic acid as radioprotectant, Ca-DTPA as radioisotope scavenger and ethanol as excipient. Radiochemical purity was assessed by radio-HPLC and was ≥95%. Incorporation yield was measured by Radio-TLC with levels of unbound ^177^Lu ≤ 0.2%.

[^177^Lu]Lu-DOTA-TOC was produced in a kit-labelling procedure by adding 240 µg DOTA-TOC dissolved in sodium ascorbate buffer (pH 5) to a vial containing 8 GBq no-carrier-added [^177^Lu]Cl3, and subsequent heating at 95°C for 30 min. The final product was formulated in physiological saline solution containing Ca-DTPA as radioisotope scavenger. Radiochemical purity was assessed by radio-HPLC and was ≥95%. Incorporation yield was measured by Radio-TLC with levels of unbound ^177^Lu ≤ 0.5%.

SPECT/CT imaging protocol:

The photo-peak was set to 208 keV with a 10 % window width, as for this setting there is a quantitative xSPECT Quant reconstruction algorithm available from Siemens Healthcare using the ordered subset conjugate gradient minimizer method (OSCGMM). The acquisition time was 5s/view resp. 10s/view for the last time point with 60 views/detector. The scanning range was defined by 3 consecutive SPECT bed positions starting from the cranium. Triple energy window (TEW) scatter correction, CT-based attenuation correction, and resolution recovery were applied for all image reconstructions. The reconstruction parameters were used as following: 48 iterations, 1 subset and Gaussian post-filter with FWHM 10.0 mm as this combination of subsets and iteration results in a good ratio between noise and resolution (1).

Bone marrow absorbed dose estimation:

The red marrow absorbed dose was estimated using the following expression:

$D=\int_{t=0}^{\infty} \frac{C(t)}{f_{M}\times f_{RM}\times\rho}dt\times\phi_{avg}\times\Delta\times k$	(1)

where *C(t)* is the activity concentration in spongiosa (Bq/ml); *f _M_* is the marrow fraction in spongiosa (0.89 and 0.90 for vertebrae and Ilium in males; 0.88 and 0.95 in females); *f _RM_* is the fraction of red marrow within the total marrow volume (0.70 and 0.48 for vertebrae and ilium, respectively); *ρ* is the red marrow density (1.03 g/ml); *φ_avg_* is the skeleton average absorbed energy fraction for red marrow (0.51 for males and 0.52 for females); Δ is the mean electron energy emitted per ^177^Lu decay (147.9 keV/decay); and *k* is a conversion factor to obtain units in Gy (5.77 x 10^-13^ Js/eV) (2, 3). To obtain *C(t)*, an exponential function was fitted to the measured mean activity concentrations in the spongiosa over time.

**Supplemental Table 1** Comparison of [^177^Lu]Lu-DOTA-TOC and [^177^Lu]Lu-DOTA-JR11 organ absorbed dose coefficients.

|  | [^177^Lu]Lu-DOTA-TOC  Median Range | [^177^Lu]Lu-DOTA-JR11  Median Range |
| --- | --- | --- |
| Kidneys; Red marrow*; Spleen | 2.01 \| 0.85-4.09; 0.21 \| 0.11-0.41; 3.78 \| 1.28-7.78 | 2.22 \| 1.08-6.67; 0.54 \| 0.32-0.66; 2.65 \| 1.04-5.89 |

Organ absorbed dose coefficients in GBq/cycle per patient expressed as median and range of all 15 patients for kidneys and spleen. *Red marrow dose could not be calculated for 9 patients due to bone metastases in the spine (7 patients) or image reconstruction issues (2 patients).

**Supplemental Fig. 1**

Post-treatment maximum intensity projection of quantitative SPECT images acquired 48 h after injection of [^177^Lu]Lu-DOTA-TOC (A) and [^177^Lu]Lu-DOTA-JR11 (B) in the same patients 10±2 weeks apart. The SUV window threshold was set at 10 for all images and scales indicate SUV values. In all 15 patients bone marrow uptake in the spine was more pronounced with [^177^Lu]Lu-DOTA-JR11 than with [^177^Lu]Lu-DOTA-TOC.

**References**

1.	Tran-Gia J, Lassmann M. Characterization of Noise and Resolution for Quantitative (177)Lu SPECT/CT with xSPECT Quant. *J Nucl Med*. 2019;60:50-59.

2.	Hough M, Johnson P, Rajon D, et al. An image-based skeletal dosimetry model for the ICRP reference adult male--internal electron sources. *Phys Med Biol*. 2011;56:2309-2346.

3.	Eckerman K, Endo A. ICRP Publication 107. Nuclear decay data for dosimetric calculations. *Ann ICRP*. 2008;38:7-96.
